# Serum Dioxin Concentrations and Quality of Ovarian Function in Women of Seveso

**DOI:** 10.1289/ehp.9667

**Published:** 2006-12-20

**Authors:** Marcella Warner, Brenda Eskenazi, David L. Olive, Steven Samuels, Sunita Quick-Miles, Paolo Vercellini, Pier Mario Gerthoux, Larry Needham, Donald G. Patterson, Paolo Mocarelli

**Affiliations:** 1 School of Public Health, University of California at Berkeley, Berkeley, California, USA; 2 Department of Obstetrics and Gynecology, University of Wisconsin Medical School, Madison, Wisconsin, USA; 3 School of Public Health, State University of New York, Albany, New York, USA; 4 Department of Obstetrics and Gynecology, Mangiagalli Hospital, University of Milan, Milan, Italy; 5 Department of Laboratory Medicine, University of Milano-Bicocca, School of Medicine, Hospital of Desio, Desio-Milano, Italy; 6 Division of Laboratory Sciences, National Center for Environmental Health, Centers for Disease Control and Prevention, Atlanta, Georgia, USA

**Keywords:** endocrine disruptor, hormones, ovary, TCDD, 2,3,7,8-tetrachlorodibenzo-*p*-dioxin

## Abstract

**Background:**

Although 2,3,7,8-tetrachlorodibenzo-*p*-dioxin (TCDD) has been associated with alterations in ovarian function and hormones in animals, it has not been studied in humans. On 10 July 1976, an explosion exposed residents of Seveso, Italy, to the highest levels of TCDD in a population. Twenty years later, we initiated the Seveso Women’s Health Study to study reproductive health.

**Objective:**

We related TCDD levels measured in sera collected near the time of explosion and ovarian function (ovarian cysts, ovarian follicles, ovulation rate, serum hormones) at follow-up.

**Methods:**

We included 363 women who were 20–40 years of age and nonusers of oral contraceptives. We examined the relationship of 1976 serum TCDD levels with ultrasound-detected ovarian follicles among 96 women in the menstrual follicular phase and serum hormone levels (estradiol, progesterone) among 129 women in the menstrual luteal phase at follow-up. Ovulation was defined by serum progesterone levels > 3 ng/mL.

**Results:**

The median serum TCDD level was 77.3 ppt, lipid-adjusted. Serum TCDD was not associated with number or size of ovarian follicles. Of women in the luteal phase, 87 (67%) ovulated. Serum log_10_TCDD was not associated with odds of ovulation [adjusted odds ratio = 0.99; 95% confidence interval (CI), 0.5 to 1.9]. Among those who had ovulated, serum log_10_TCDD was not associated with serum progesterone [adjusted beta (adj-β ) = −0.70; 95% CI, −2.4 to 1.0] or estradiol (adj-β = −1.81; 95% CI, −10.4 to 6.8).

**Conclusions:**

We found no clear evidence that 1976 TCDD exposure was associated with ovarian function 20 years later in women exposed to relatively high levels in Seveso, Italy.

2,3,7,8-Tetrachlorodibenzo-*p*-dioxin (TCDD) is a widespread environmental contaminant (Zook and Rappe 1994) and known endocrine disruptor (Birnbaum and Tuomisto 2000). In animal studies, significant effects on ovarian function and steroid levels have been reported with both *in utero* and postnatal TCDD exposure. Studies in rats and monkeys suggest that TCDD may affect ovarian function directly or indirectly via the pituitary (Gao et al. 2000; Li et al. 1995b; Moran et al. 2001, 2002).

*In utero* and lactational TCDD exposure in rats has been associated with reduced ovarian weight and decreased numbers of corpora lutea and preantral and antral follicles (Flaws et al. 1997; Gray and Ostby 1995; Heimler et al. 1998). Postnatal TCDD exposure in rats has been associated with reduced ovarian weight gain, ovulation rate, and numbers of corpora lutea and follicles, as well as inhibition of follicular rupture, morphologic changes in the ovary, and altered cyclicity with disruption of the estrous cycle (Gao et al. 1999; Kociba et al. 1976; Li et al. 1995a, 1995b; Roby 2000, 2001; Salisbury and Marcinkiewicz 2002; Silbergeld and Mattison 1987; Son et al. 1999; Umbreit et al. 1987). Although TCDD does not increase apoptosis of follicles (Heimler et al. 1998), it appears to slow follicular maturation (Mattison 1980; Silbergeld and Mattison 1987; Son et al. 1999).

Alterations in steroid levels have also been noted with TCDD exposure. *In utero* and lactational exposure in rats was associated with decreased estradiol (Chaffin et al. 1996). In rats, increased estradiol, reduced follicle stimulating hormone (FSH) and luteinizing hormone, and no change in progesterone were found during proestrous (Gao et al. 1999; Li et al. 1995a). Similar outcomes have been reported in primates, including decreases in estradiol and progesterone (Allen et al. 1979; Barsotti et al. 1979; Guo et al. 1999; Moran et al. 2001). In human luteinizing granulosa cells, TCDD decreased estradiol (Enan et al. 1996), but did not alter progesterone production (Moran et al. 2000). The above evidence suggests that TCDD could alter human ovarian function, including steroidogenesis and ovulation.

In a case report, a woman with extremely high serum TCDD (144,000 ppt) had amenorrhea and decreased serum estradiol and progesterone levels (Geusau et al. 2001). To our knowledge, no epidemiologic studies have examined the potential effects of TCDD exposure on quality of human ovarian function.

On 10 July 1976, as a result of a chemical explosion, residents of Seveso, Italy, experienced the highest levels of TCDD exposure known in a residential population (Mocarelli et al. 1988). Twenty years later, we initiated the Seveso Women’s Health Study (SWHS) to examine the relation of TCDD levels in serum collected soon after the explosion with reproductive health. We previously reported that serum TCDD levels were associated with an increase in risk for earlier menarche [hazard ratio = 1.2; 95% confidence interval (CI), 0.98 to 1.6] among women who were < 5 years of age at the time of the explosion (Warner and Eskenazi 2005), an average increase in menstrual cycle length of almost a full day but only among those who were pre-menarcheal at exposure (Eskenazi et al. 2002), and a nonmonotonic dose-related association with earlier onset of natural menopause up to a TCDD level of 100 ppt, but not above (Eskenazi et al. 2005). In the present study, we examined the relationship of serum TCDD levels measured in serum collected near the explosion with quality of ovarian function, including ovarian follicles, functional ovarian cysts, ovulation rate, and serum hormone levels.

## Materials and Methods

### Study population

The SWHS is the first comprehensive epidemiologic study of the reproductive health of a female population exposed to TCDD. Women eligible for SWHS were 1 month to 40 years of age in 1976, had resided in one of the most highly contaminated areas based on surface soil TCDD levels (Zone A or B), and had adequate stored sera collected soon after the explosion (Eskenazi et al. 2000). Recruitment took place from March 1996 through July 1998. Of 1,271 eligible women, 17 could not be contacted, and 33 had died or were too ill to participate. Of the 1,221 women contacted, 981 (80%) agreed to participate. Eligible for the quality of ovarian function analysis were 363 women who were 20–40 years of age and not using oral contraceptives at follow-up. The functional ovarian cyst analysis included the 310 women who underwent ultrasound. The ovarian follicle analysis was limited to the 96 women who were in the pre-ovulatory window of the follicular phase of the menstrual cycle (determined by subtracting 14–18 days from self-report of usual cycle length and date of last menstrual period) at the time of ultrasound. The hormone analysis was limited to the 129 women in the luteal phase (last 14 days) of their menstrual cycle at the time of the blood draw, based on self-report of usual cycle length and date of last menstrual period.

### Procedure

This study was approved by the institutional review boards of the participating institutions. Details of the study are presented elsewhere (Eskenazi et al. 2000). Briefly, after participants gave written informed consent, they underwent a fasting blood draw and were interviewed by a trained nurse-interviewer who was blinded to TCDD level and residence of the woman. Information was collected during the interview about demographic characteristics, personal habits, and occupational, menstrual, reproductive, and medical histories. Medical records were requested for all gynecologic treatments or conditions. After the interview, women who were ≤ 50 years of age at interview and who were still menstruating were invited to undergo a gynecologic examination and transvaginal ultrasound, and to complete a menstrual cycle diary for 3 months. Gynecologists at the University of Milan, Mangiagalli Hospital, and for a few cases at the Hospital of Desio conducted the examinations and ultrasounds. The ultrasounds were recorded on videotape, ovaries were photographed, and a structured codeable data form was completed at the time of the examination of each ovary with the notation of specific items such as ovarian cysts. The transvaginal ultrasound videotapes were reviewed by a second gynecologist (D.L.O.), the number of follicles was counted, and the diameter of all follicles > 10 mm was recorded.

### Laboratory analyses

#### Serum TCDD analysis

TCDD was measured in archived sera by high-resolution gas chromatography/high-resolution mass spectrometry methods (Patterson et al. 1987). Values are reported on a lipid-weight basis in parts per trillion (Akins et al. 1989).

Details of serum sample selection are presented elsewhere (Eskenazi et al. 2000). For the 363 women eligible for this analysis, we measured TCDD in sera collected between 1976 and 1977 for 330 (90.9%) women; between 1978 and 1982 for 25 (6.9%) women; and between 1996 and 1997 for 8 (2.2%) women whose earlier samples had insufficient volume. For nondetectable values (*n* = 29), a serum TCDD level equal to one-half the detection limit was assigned (Hornung and Reed 1990). For women with detectable post-1977 TCDD measurements (≥ 10 ppt), the TCDD exposure level was back-extrapolated to 1976 using the first-order kinetic model (Pirkle et al. 1989) for women who were > 16 years of age in 1976 (*n* = 2) or the Filser model (Kreuzer et al. 1997) otherwise (*n* = 25). For women with post-1977 TCDD measurements that were detectable but < 10 ppt (*n* = 3), the measured value was used for analysis. The study median serum sample weight was 0.65 g, and the median limit of detection was 18.8 ppt, lipid-adjusted.

#### Serum hormone analyses

Serum estradiol and progesterone levels were measured in blood collected at the time of interview for 129 women. We classified ovulation status (yes/no as to whether she had ovulated) for each woman based on a serum progesterone level > 3 ng/mL. Serum hormone analyses were performed at the Hospital of Desio. Serum estradiol measurements were made in duplicate using the microparticle enzyme immunoassay (Abbott Axsym System; Abbott Laboratory, Abbott Park, IL, USA), and serum progesterone measurements were made using the chemiluminescence immunoassay (Advia Centaur System; Bayer, East Walpole, MA, USA).

### Statistical analyses

#### Ovarian follicles and functional ovarian cysts

For the ovarian follicle analysis, we considered serum TCDD levels both as a continuous (log_10_ TCDD) and a categorical variable. The lowest TCDD cut-point was first set at ≤ 20.0 ppt, and then the remainder was divided into two equal size groups (20.1–100, > 100 ppt). We selected 20 ppt (body burden ≈4 ng/kg) as the cut-point because this was the average TCDD level of 1976 serum pools collected from Italian women living in an unexposed area (Eskenazi et al. 2004). The prevalence of ovarian follicles > 10 mm was considered a dichotomous outcome (any/none). To evaluate the relationship between serum TCDD and prevalence of ovarian follicles, we performed logistic regression. The total number of ovarian follicles, number of ovarian follicles > 10 mm in diameter, and diameter of dominant ovarian follicle were all examined as continuous dependent variables using multiple linear regression models.

Covariates were considered for the multivariate regression analysis if they had been reported in previous literature to be related to ovarian follicles or functional cysts (Christensen et al. 2002; Holt et al. 2003, 2005). We considered the following potential covariates: age at ultrasound, age at explosion, age at menarche, menarche status at explosion, marital status, parity, gravidity, age at last birth, lactation history, current body mass index (BMI), smoking, and education. We also considered effect modification by menarche status at explosion. Covariates were kept in the multivariate model if they were statistically significant (*p* < 0.10).

For the functional ovarian cyst analysis, the number of cases limited analyses to descriptive statistics.

#### Serum hormones and ovulation status

For the serum hormone analysis, we considered serum TCDD levels both as a continuous (log_10_TCDD) and a categorical variable. Similar to the ovarian follicle analysis, the lowest TCDD cut point was first set at ≤ 20.0 ppt, and then the remainder was divided into three equal size groups (20.1–77.0, 77.1–212.0, > 212 ppt). To evaluate the relationship between serum TCDD and ovulation status, we performed logistic regression. For the subset of women whom we judged to have ovulated (progesterone level > 3 ng/mL), serum estradiol and progesterone levels were examined as a continuous dependent variable using multiple linear regression models. Analyses were performed using STATA 8.0 (StataCorp., College Station, TX, USA). All *p*-values are two-sided.

We considered covariates for the multivariate analyses if they had been reported in previous literature to be related to ovulation status or hormone levels (Windham et al. 2002). We considered the following as potential covariates: age at blood draw, age at explosion, age at menarche, menarche status at explosion, marital status, parity, gravidity, history of abortion (voluntary or spontaneous), mid-luteal phase at time of blood draw, current BMI, smoking, alcohol consumption, coffee consumption, tea consumption, soda consumption, current physical activity, and oral contraceptive use (ever/never, total years, time since last use). Covariates were kept in the multivariate model if they were statistically significant (*p* < 0.10). We also considered effect modification by menarche status at explosion.

Because square-root transformation improved the normality of serum estradiol slightly, linear regression models with estradiol as the dependent variable were run with and without square-root transformation. The results were not different, so we report only the untransformed results. All final models were rerun excluding women who reported stopping oral contraceptive use in the preceding 12 months (*n* = 11 for ovulation status, *n* = 4 for serum hormones). Again, the results were not different; therefore we report only the full sample results.

## Results

Characteristics of the 363 women eligible for the analysis are presented in Table 1. The average age (± SD) at interview of the 363 women was 31.3 ± 5.3 years. All women were Caucasian, 75% had finished more than the required amount of education, 67% had ever married, 17% were overweight or obese (BMI > 25 kg/m^2^), 58% had ever used oral contraceptives, 61% had never smoked, 88% currently drank coffee, 53% were parous, and 46% had reached menarche before the explosion. Overall, the median lipid-adjusted serum TCDD level for the 363 women was 77.3 ppt (interquartile range, 33–214 ppt; range, 2.8–17,300 ppt).

### Ovarian follicles and functional ovarian cysts

Of the 96 women in the pre-ovulatory window of the follicular phase of their menstrual cycle at the time of ultrasound, 94 (98%) had any follicles visualized in the left and/or right ovary (mean ± SD = 9.5 ± 5.2 follicles; range, 1–24). Of these, 65 ± 67.7% women had at least one follicle (range, 1–4) > 10 mm in diameter. The median size of the dominant follicle ranged from 10 to 28 mm (median = 16 mm). The median lipid-adjusted serum TCDD level for the 65 women with at least one follicle > 10 mm was 80.5 ppt (range, 4–2,730), nonsignificantly (*p* = 0.76) lower than for the 31 women with no follicles > 10 mm in diameter (median = 90.5 ppt; range, 5.5–4,730).

As presented in Table 2, after controlling for age and quality of ultrasound, a 10-fold increase in TCDD (log_10_TCDD) was not associated with odds of having any follicles > 10 mm [adjusted odds ratio (AOR) = 0.99; 95% CI, 0.4 to 2.2; *p* = 0.99]. When serum TCDD levels were categorized, compared with the lowest exposure group, women were more likely to have any follicles > 10 mm, but not significantly, and there was no evidence of a dose response (test for trend: *p* = 0.92). After adjusting for covariates, relative to women with TCDD levels ≤ 20.0 ppt, the odds of having any follicles > 10 mm among women with TCDD levels from 20.1 to 100 ppt and > 100 ppt were 2.90 (95% CI, 0.8 to 10.8; *p* = 0.11) and 1.45 (95% CI, 0.4 to 5.5; *p* = 0.59), respectively.

In regression analysis, total number of ovarian follicles was not related to serum TCDD level. After adjusting for age and quality of ultrasound, a 10-fold increase in TCDD (log_10_TCDD) was not associated with a change in total follicles [adjusted β (adj-β )= 0.49; 95% CI, −1.1 to 2.1; *p* = 0.54]. When TCDD levels were categorized, there was still no evidence of a dose response with total follicles (test for trend: *p =* 0.80). The size of the dominant follicle was also not related to serum TCDD level either as a continuous (adj-β = 0.21; 95% CI, −2.9 to 3.4; *p* = 0.89) or a categorical variable (*p* = 0.99).

In total, 8 of 310 women had a functional ovarian cyst (7 with follicular, 1 with luteal) diagnosed at ultrasound. The median serum TCDD levels for the 8 functional cyst cases (median = 82.3 ppt) was only slightly higher than noncases (*n* = 302, median = 78.0 ppt), but not significantly (*p* = 0.69).

### Serum hormones and ovulation

Of the 129 women who were in the luteal phase of their menstrual cycle at blood draw, 87 (67%) were classified as having ovulated (> 3 ng/mL progesterone). Lipid-adjusted serum TCDD levels for the 87 women who had ovulated (median = 112.0 ppt) were not significantly different from the levels of 42 women who had not ovulated (median = 107.1 ppt) (*p* = 0.71).

As presented in Table 3, after controlling for age, mid-luteal phase, and oral contraceptive use in the preceding year, a 10-fold increase in TCDD (log_10_TCDD) was not associated with a reduced odds of ovulation (AOR = 0.99; 95% CI, 0.5 to 1.9; *p* = 0.97). When serum TCDD levels were categorized, compared with the lowest exposure group, women were less likely to ovulate, but not significantly, and there was no evidence of a dose response (test for trend: *p* = 0.93). After adjusting for covariates, relative to women with TCDD levels ≤ 20.0 ppt, the odds of ovulation among women with TCDD levels from 20.1 to 77.0 ppt, 77.1 to 212.0 ppt, and > 212.0 ppt were 0.59 (95% CI, 0.1 to 2.5; *p* = 0.48), 0.66 (95% CI, 0.2 to 2.8; *p* = 0.57), and 0.73 (95% CI, 0.2 to 3.4; *p* = 0.69), respectively.

When we repeated the analysis in the subset of 74 women who were in the mid-luteal phase of their menstrual cycle at blood draw, the results were similar (Table 3). After controlling for age and oral contraceptive use in the preceding year, log_10_TCDD was not associated with a decreased odds of ovulation (AOR = 1.03; 95% CI, 0.4 to 2.7; *p* = 0.96). When serum TCDD levels were categorized, there was still no evidence of a dose response (test for trend: *p* = 0.95). After adjusting for covariates, relative to women with TCDD levels ≤ 20.0 ppt, the odds of ovulation among women with TCDD levels from 20.1 to 77.0 ppt, 77.1 to 212.0 ppt, and > 212.0 ppt were 0.71 (95% CI, 0.1 to 6.5; *p* = 0.76), 1.23 (95% CI, 0.1 to 10.9; *p* = 0.85), and 0.74 (95% CI, 0.1 to 7.1; *p* = 0.80), respectively.

Among the 87 women who were classified as having ovulated, mean serum levels of progesterone and estradiol were 11.0 ± 5.8 ng/mL and 78.4 ± 29.1 pg/mL, respectively. Serum progesterone levels were significantly higher among women in the mid-luteal phase of their menstrual cycle at time of blood draw, and both progesterone and estradiol levels were lower among older women, but not significantly. In regression analysis of women who had ovulated (Table 4), after controlling for age and mid-luteal phase, a 10-fold increase in TCDD (log_10_TCDD) was not associated with serum progesterone (adj-β = −0.70; 95% CI, −2.4 to 1.0; *p* = 0.42) or serum estradiol (adj-β = −1.81; 95% CI, −10.4 to 6.8; *p* = 0.68). As presented in Table 4, when serum TCDD levels were categorized, compared with the lowest exposure group, progesterone and estradiol levels both were decreased, but not significantly, and there was no evidence of a dose response for either hormone (test for trend: progesterone *p =* 0.51; estradiol *p* = 0.47).

When we repeated the analysis in the -subset of 55 women who had ovulated and were in the mid-luteal phase of their menstrual cycle at blood draw, the results were similar (Table 4). After controlling for age, log_10_TCDD was not associated with progesterone (adj-β = −0.84; 95% CI, −3.7 to 2.0; *p* = 0.56) or estradiol (adj-β = −3.11; 95% CI, −14.1 to 7.8; *p* = 0.57). When serum TCDD levels were categorized, the decrease in progesterone and estradiol was larger, but there was still no evidence of a dose-response for either hormone (test for trend: progesterone *p =* 0.57; estradiol *p* = 0.25). No significant interaction was found between menarche status at exposure and TCDD for progesterone (*p* = 0.23) or estradiol (*p* = 0.96).

The results did not change when women who reported cessation of oral contraceptives in the preceding 12 months (*n* = 11 in ovulation models, *n* = 4 in progesterone and estradiol models) were excluded (data not shown).

## Discussion

To our knowledge, this is the first epidemiologic study to examine the relation of individual serum TCDD levels and quality of ovarian function in a highly exposed population. The results of this study of women residing in Seveso, Italy, in 1976 at the time of an explosion, which released high levels of TCDD, provide no clear evidence of an association of exposure with quality of ovarian function approximately 20 years later. TCDD levels measured in serum collected near the time of exposure were not associated with number or size of ultrasound-detected ovarian follicles, ovulation status, or serum hormone levels at follow-up.

This study also has several limitations that should be considered. Although an association between TCDD and ovarian function is biologically plausible based on animal evidence, it is possible that the women included in the current study were not exposed during a critical period of development. In most of the above-described animal studies, animals were exposed prenatally whereas SWHS women were exposed postnatally. Another limitation is that we did not measure FSH and therefore we were not able to examine the role of TCDD on ovarian reserve. However, we previously reported a nonmonotonic dose-related association with increasing risk of earlier menopause up to about 100 ppt TCDD, but not above, among women in SWHS—suggesting the possibility of a relation of TCDD on ovarian reserve.

In addition, phase of cycle may have been misclassified for some women because the day of cycle was based on self-report of menstrual cycle length and date of last menstrual period. We were able to collect only one serum hormone measure during the menstrual cycle and may have missed an effect of TCDD by measuring over too wide a range in the menstrual cycle, as evidenced by the somewhat stronger finding of decrease in hormone levels when the analysis was limited to women in the mid-luteal phase. However, this study did measure serum hormone levels directly, rather than measuring urinary metabolites, which may vary by woman depending on intrinsic and extrinsic factors (Windham et al. 2002).

Another limitation of the study is that the lowest exposure group (≤ 20.0 ppt) had relatively high serum TCDD levels compared with the contemporary levels we have reported for this area (~ 2 ppt) (Warner et al. 2004). Also, although the explosion resulted in exposure specifically to TCDD, pooled serum samples collected in 1976 from females who resided in the unexposed area showed substantial background dioxin toxic equivalents (TEQ) exposure (average = 100.4 ppt total TEQ, with TCDD contributing 20.2 ppt TEQ and analytes other than TCDD contributing 80.2 ppt TEQ) during this time period (Eskenazi et al. 2004). Therefore, SWHS participants with TCDD levels < 20 ppt might still have had substantial total TEQ exposure. Because we measured only TCDD in this study due to the small sample volume available (0.65 grams), our results may underestimate an effect due to dioxin TEQ exposure. However, an important advantage of this study is that we were able to measure TCDD levels in individual serum samples collected near the time of exposure, and there was a wide range of exposure.

In summary, we found no clear evidence of an association of TCDD exposure measured in serum collected near the explosion on quality of ovarian function 20 years later, including ovarian follicles, ovulation status, and serum hormones. The women in this study experienced substantial TCDD exposure during the postnatal developmental period. Animal evidence suggests that *in utero* and lactational TCDD exposure may have more significant effects on ovarian function (Benedict et al. 2000; Heimler et al. 1998); therefore, continued follow-up of the younger women in the SWHS cohort as well as the female offspring of the SWHS cohort is essential.

## Figures and Tables

**Table 1:** Select characteristics of the subsample of 363 women (≤ 40 years of age who were not currently on oral contraceptives), SWHS, 1996–1998.

Characteristic	No. (%)
Total	363 (100)
Age at interview (years)	
20–28	112 (30.9)
29–33	118 (32.5)
34–40	133 (36.6)
Education	
≤ Required	93 (25.6)
Intermediate/professional	147 (40.5)
≥ High school	123 (33.9)
Married	
Never	119 (32.8)
Ever	244 (67.2)
Current BMI	
Underweight	32 (8.8)
Normal	268 (73.8)
Overweight	46 (12.7)
Obese	17 (4.7)
Oral contraceptive use	
Never	152 (41.9)
Ever	211 (58.1)
Cigarette smoking	
Never	223 (61.4)
Former	53 (14.6)
Current	87 (24.0)
Parity	
0	171 (47.1)
1	88 (24.2)
≥ 2	104 (28.7)
Lactation history (months)	
0	194 (53.4)
1–6	105 (28.9)
> 6	64 (17.6)
Menarche before explosion	
No	195 (53.7)
Yes	168 (46.3)

**Table 2:** Adjusted odds ratio (AOR) and 95% CI for any follicles with serum TCDD level among women in the follicular phase (*n* = 96), SWHS, 1996–1998.

TCDD (ppt)	Any/total (%)	AOR^a^ (95% CI)
log TCDD	65/96 (67.7)	0.99 (0.4 to 2.2)
≤ 20.0	7/13 (53.8)	1.0
20.1–100	31/41 (75.6)	2.90 (0.8 to 10.8)
> 100	27/42 (64.3)	1.45 (0.4 to 5.5)

a Adjusted for age at ultrasound and quality of ultrasound.

**Table 3:** AOR (95% CI) for ovulation with serum TCDD level among women in the luteal phase (*n* = 129) and the mid-luteal phase (*n* = 74), SWHS, Italy, 1996–1998.

	Women in luteal phase		Women in mid-luteal phase	
TCDD (ppt)	Ovulate/total (%)	AOR (95% CI)^a^	Ovulate/total (%)	AOR (95% CI)^b^
log TCDD	87/129 (67)	0.99 (0.5 to 1.9)	55/74 (74)	1.03 (0.4 to 2.7)
≤ 20.0	10/14 (71)	1.00	5/7 (71)	1.00
20.1–77.0	24/38 (63)	0.59 (0.1 to 2.5)	15/22 (68)	0.71 (0.1 to 6.5)
77.1–212.0	26/39 (67)	0.66 (0.2 to 2.8)	17/21 (81)	1.23 (0.1 to 10.9)
> 212	27/38 (71)	0.73 (0.2 to 3.4)	18/24 (75)	0.74 (0.1 to 7.1)

a Adjusted for age, mid-luteal phase, and oral contraceptive use in preceding year.

b Adjusted for age and oral contraceptive use in preceding year.

**Table 4:** Adj-β (95% CI) for serum progesterone and estradiol per change in serum TCDD levels among women who had ovulated and were in the luteal phase (*n* = 87) and the mid-luteal phase (*n* = 55), SWHS, Italy, 1996–1998.

	Women in luteal phase		Women in mid-luteal phase	
Hormone, TCDD	No. (%)	Adj-β a (95% CI)	No. (%)	Adj-β b (95% CI)
Progesterone				
log TCDD	87 (100)	−0.70 (−2.4 to 1.0)	55 (100)	−0.84 (−3.7 to 2.0)
≤ 20.0	10 (11.5)	1.00	5 (9.1)	1.00
20.1–77.0	24 (27.6)	0.02 (−4.8 to 4.8)	15 (27.3)	−3.12 (−11.3 to 5.0)
77.1–212.0	26 (29.9)	−2.35 (−6.9 to 2.2)	17 (30.9)	−4.93 (−12.7 to 2.9)
> 212	27 (31.0)	−0.77 (−5.4 to 3.9)	18 (32.7)	−3.09 (−11.3 to 5.2)
Estradiol				
log TCDD	87 (100)	−1.81 (−10.4 to 6.8)	55 (100)	−3.11 (−14.1 to 7.8)
≤ 20.0	10 (11.5)	1.00	5 (9.1)	1.00
20.1–77.0	24 (27.6)	−3.01 (−21.4 to 15.3)	15 (27.3)	−10.51 (−37.0 to 16.0)
77.1–212.0	26 (29.9)	−10.90 (−29.6 to 7.8)	17 (30.9)	−16.02 (−43.6 to 11.6)
> 212	27 (31.0)	−5.63 (−22.5 to 11.2)	18 (32.7)	−15.89 (−39.6 to 8.0)

a Adjusted for age and mid-luteal phase.

b Adjusted for age.
